# Nonattainability of the Fragility Index

**DOI:** 10.7759/cureus.108357

**Published:** 2026-05-06

**Authors:** Thomas F Heston

**Affiliations:** 1 Medical Education and Clinical Sciences, Washington State University Spokane, Spokane, USA; 2 Family Medicine, University of Washington, Spokane, USA

**Keywords:** clinical trial methodology, contingency tables, domain incompleteness, fisher's exact test, fragility index, partial function

## Abstract

Background: The fragility index (FI) is intended to quantify how many outcome changes would be required to convert a statistically significant two-arm trial result into a nonsignificant one. A reliable statistical metric should produce a result for every valid case it evaluates. This study examined whether a fragility value is always attainable for every statistically significant trial result.

Methods: FI was analyzed as follows: baseline significance was required (p < 0.05), one-way movement only, and outcome changes were restricted to converting a nonevent to an event in the arm with fewer events, while keeping the arm size fixed. Nonattainability was assessed by determining whether valid 2×2 tables exist for which no finite FI can be obtained under these rules. Evidence is provided through formal counterexamples, complete enumeration of all valid nondegenerate 2 × 2 tables up to total sample size N = 60, and empirical evaluation of published two-arm trials with binary outcomes.

Results: Valid baseline-significant 2 × 2 tables exist for which FI is not attainable. A simple counterexample is {3,0,4,11}: baseline two-sided Fisher's exact p = 0.0429, the arm with fewer events is uniquely identified, but that arm has no nonevents available for the required toggle; thus, no legal FI path exists. Enumeration revealed that unattainable cases first appeared at N = 18 and then recurred at every larger sample size through N = 60; by N = 60, a total of 2,390 of 20,774 evaluable baseline-significant tables were unattainable (11.5%). In an empirical dataset of published trials, 2 of 82 baseline-significant evaluable trials (2.4%) were not attainable.

Conclusions: The FI is not universally attainable. This is a structural property of the FI algorithm, confirmed by mathematical proof, a complete table enumeration, and published trial data.

## Introduction

The fragility index (FI) was introduced as the minimum number of patients whose outcome status would need to change from a nonevent to an event to convert a statistically significant trial result into a nonsignificant one [1]. As originally defined, the FI was calculated only for trials with statistically significant dichotomous results, and the event changes were applied to the arm with fewer events while preserving arm size. This made FI an intuitively appealing patient-level summary of how easily statistical significance could be lost. FI has since been widely used across clinical specialties because it expresses threshold stability in terms of patient outcomes rather than abstract statistical quantities, with applications spanning cardiovascular, pediatric, surgical, and rheumatology research [2-5].

Prior scholarly attention has focused on interpretation and context: whether FI correlates too closely with the p-value to add independent information [6], whether the raw count should be normalized by sample size [7], and whether the toggling direction should reflect clinical plausibility rather than algorithmic convenience [8]. These are legitimate concerns, but they are all questions about how FI should be interpreted or reported, not whether it can always be computed.

The question addressed here is narrower and more fundamental: does the FI always produce a finite value on the class of valid 2 × 2 tables to which it is applied? It is proposed that attainability - the property of producing a result for every valid input - is a logical prerequisite for any measure proposed as a universal reporting standard. As past critiques of p-value thresholds have demonstrated, adopting rigid standards without fully understanding their functional limitations can lead to severe distortions in how scientific evidence is evaluated and reported [9]. Whether the FI is universally attainable has not been formally examined. This question has three separable components: a formal claim established or refuted by a single counterexample, a descriptive claim about how often such cases arise across the enumerated table space, and a demonstration of nonattainability in published clinical trials.

This is not a mathematical abstraction - it is the same standard that applies to any routine statistical metric. For example, Fisher’s exact test is a commonly applied method for evaluating 2 x 2 contingency tables [10]. If Fisher's exact test simply returned no value for an undetermined subset of valid tables, it would never be considered a reliable statistical tool. The FI should adhere to this same standard.

This question has practical urgency. Calls for the routine integration of FI into clinical trial reporting have increased substantially, with some authors also recommending its inclusion in clinical guideline development [11,12]. Any such mandate implicitly assumes that every valid baseline-significant trial can be assigned a finite fragility value. Whether that assumption holds has not been formally tested. Modifications to the FI have been proposed, but have not been fully standardized or recognized [13-15]. Therefore, this analysis evaluated whether the original published definition of the FI always produces an attainable result [1].

A working draft for this manuscript has been deposited on the Research Square preprint server [16].

## Materials and methods

Definition of the FI

For a valid 2 × 2 table {a, b, c, d}, where a and c are events and b and d are nonevents, the FI was defined as follows: (a) the baseline table must be statistically significant according to a two-sided Fisher's exact test, with p < 0.05; (b) identify the arm with fewer events; (c) convert one nonevent to an event in that same arm, preserving the arm total; (d) the two-sided Fisher’s exact p-value was recalculated after each such change; (e) the FI is the minimum number of allowable changes required to make the result nonsignificant (p >= 0.05); (f) if no finite sequence of allowable changes exists, the FI is not attainable.

Tables with tied event counts were excluded from the primary analysis because the definition specifies the arm with fewer events and does not provide a tie-breaking rule. Contingency tables are referred to by the standard nomenclature {a, b, c, d}, where a = events in arm A, b = nonevents in arm A, c = events in arm B, and d = nonevents in arm B. Note that the FI definition does not clearly state what to do when outcome events are tied (a = c). This is the original and established definition of FI [1].

Definition of nonattainability

An algorithm is considered not universally attainable if there exists at least one valid input for which no finite output can be produced under its own rules [17]. In mathematical terms, this represents a partial function - an algorithm that fails to assign a value to every possible valid input. Therefore, the existence of a single valid baseline-significant 2 × 2 table with an unattainable FI is sufficient to establish that the metric is not universally attainable. All analyses used the original definition and arm selection rule [1]. The FI was calculated using the Fragility Metrics Toolkit [18], which explicitly returns 'Not Attainable' for tables where no finite FI exists. For external reproducibility checks, counterexamples were also entered into the ClinCalc Fragility Index Calculator [19], which applies the same Walsh-style iterative Fisher exact test but reports nonattainability only as a generic calculation error message.

Formal counterexample analysis

The constructed tables were examined to determine whether valid baseline-significant examples exist for which the FI procedure cannot produce a finite value. For each counterexample, the baseline Fisher's exact p-value was computed, and every allowable step was evaluated.

Complete enumeration

To determine whether nonattainability is isolated or recurrent, all valid nondegenerate 2 × 2 tables were enumerated for total sample sizes N = 2 through N = 60. For each N, every integer-valued table {a,b,c,d} satisfying a + b + c + d = N was generated. A table was retained for analysis if it met all four of the following criteria: each arm contained at least one subject (a + b > 0 and c + d > 0); each outcome column was present (a + c > 0 and b + d > 0); the baseline two-sided Fisher’s exact *p* value was less than 0.05; and event counts were not tied (a ≠ c). FI was then evaluated on all the retained tables. For each N, the following were recorded: total number of valid tables, number of baseline-significant tables, number with attainable FI, number with unattainable FI, and number excluded because event counts were tied. This enumeration was not intended as a prevalence estimate for published trials. Its purpose was structural: to determine whether unattainability recurs across the valid table space and to characterize the mechanisms by which it arises.

Two predictors of FI nonattainability were evaluated across the enumerated table set (N = 2-60): allocation imbalance, operationalized as the ratio of arm sizes (n1/n2), and total sample size. Spearman's rank correlations were computed with the individual table as the unit of analysis. The predictors were selected to test whether nonattainability scales with the structural geometry of the table (imbalance) or with raw sample size.

Empirical dataset

An empirical convenience dataset of 143 published two-arm trials with binary outcomes was assembled from prior fragility-index publications and recent high-impact clinical trials identified through PubMed (2014-2025), spanning predominantly cardiovascular, oncology, and critical care domains. Inclusion required a two-arm design, a binary primary outcome, and reported event counts in both arms. The dataset was not assembled as a systematic review and is not intended as a prevalence estimate. Only baseline-significant, nontied tables were counted as evaluable FI cases.

Data availability

The R code, data, and draft are archived on the Zenodo repository [20].

## Results

Mathematical proof

Theorem

The FI is not universally attainable.

Proof

A metric is not universally attainable if there exists at least one valid input for which the algorithm produces no finite value.

Consider the 2 × 2 table {3, 0, 4, 11}. The baseline two-sided Fisher's exact test yields p = 0.0429, confirming that the contingency table is valid for the FI algorithm to be applied. Arm A has fewer events than Arm B does (3 < 4); thus, all toggles must occur in Arm A. However, Arm A has zero nonevents (b = 0), so no legal move exists. The algorithm cannot begin, and no finite FI value can be obtained. This valid, baseline-significant table demonstrates that the FI is not universally attainable.

A second counterexample, {9,1,10,90}, demonstrates that nonattainability extends beyond zero-nonevent cases. The two-sided Fisher's exact *p*-value is <0.0001, indicating that the contingency table is valid for the FI algorithm. Arm A has fewer events (9 < 10), so all changes must occur in arm A. Only one legal change is possible because arm A has a single nonevent. After that change, yielding {10,0,10,90}, the result remains highly significant (p < 0.0001). No further changes are possible. FI is unattainable, indicating that the metric does not yield a value for every valid input.

A third counterexample, {9,35,8,8}, isolates the algorithm's arm-selection behavior without relying on cells with a value of 0 or 1. The two-sided Fisher's exact p-value is 0.0487, making it a valid contingency table for the FI algorithm to be applied. Arm A has 9 events and 35 nonevents, and arm B has 8 events and 8 nonevents. Arm B has fewer events in absolute terms (8 < 9), so all FI changes must occur in arm B. After each allowable toggle, arm B's event rate increases from 50% toward 100%, widening the disparity between the arms. Each successive toggle moves the two-sided Fisher's exact p-value further from the nonsignificance boundary rather than toward it. All 8 available nonevents in arm B were exhausted, and the p-value did not reach 0.05. The full toggle trajectory was: baseline {9,35,8,8} p = 0.0487; toggle 1 {9,35,9,7} p = 0.0116; toggle 2 {9,35,10,6} p = 0.0039; toggle 3 {9,35,11,5} p = 0.0012; toggle 4 {9,35,12,4} p = 0.0002; toggles 5-8 all p < 0.0001, with arm B exhausted at toggle 8 without ever crossing the 0.05 threshold. FI is unattainable, further demonstrating that the metric does not yield a value for every valid input.

Enumeration results

Complete enumeration of all valid nondegenerate 2 × 2 tables up to a total sample size of N = 60 revealed that unattainable FI cases are recurrent rather than isolated.

No unattainable cases were found for N < 18. The first unattainable cases appeared at N = 18, where 2 of 316 evaluable baseline-significant tables were unattainable. Thereafter, unattainable cases recurred at every larger sample size examined; representative results are shown in Table 1. Among the evaluable tables (attainable plus unattainable, excluding ties), FI was not attainable in 28,982 out of 296,192 tables (9.8%). The unattainability rate peaked at 11.5% at N = 60. The increase from N = 18 to N = 60 was monotonic with a decelerating slope; characterization of asymptotic behavior beyond N = 60 was not undertaken. Representative results are shown in Table 1.

**Table 1:** Selected results from complete enumeration by total sample size. For each total sample size shown, the table reports the number of baseline-significant nondegenerate 2 × 2 tables, the number with an attainable and unattainable fragility index (FI), the number excluded because event counts were tied, and the resulting unattainability rate calculated as unattainable/(attainable + unattainable).

N	Total significant	FI attainable	FI unattainable	Ties excluded	Unattainability rate
18	324	314	2	8	0.60%
20	460	442	8	10	1.80%
25	1060	998	36	26	3.50%
30	2010	1850	112	48	5.70%
40	5404	4856	432	116	8.20%
50	11578	10220	1142	216	10.10%
60	21112	18384	2390	338	11.50%

To determine whether nonattainability is an artifact of zero-cell tables, which Walsh's single-arm, single-direction algorithm cannot escape by construction, the minimum cell value was recorded for each unattainable table. If zero-cell tables were the sole mechanism, all unattainable cases would have a minimum cell value of zero. In the enumerated tables (N = 2-60), the minimum cell value ranged from 0 to 8, as shown in Table 2.

**Table 2:** Minimum cell count of tables with an unattainable FI. This table shows, across all enumerated unattainable cases from N = 2-60, the minimum cell value present in each table and its frequency to assess whether FI nonattainability is limited to zero-cell tables or also occurs when all cells are nonzero. FI: fragility index

Minimum cell value	Count	Percent
0	12,278	42.36
1	7178	24.77
2	4284	14.78
3	2602	8.98
4	1492	5.15
5	748	2.58
6	286	0.99
7	102	0.35
8	12	0.04
Total	28,982	100

The minimum cell value of zero accounted for 42.4% of the unattainable cases, which is consistent with the expected dead end in which the toggling arm is exhausted before significance is lost. The remaining 57.6% of unattainable cases had no zero cells. In these tables, the toggling arm contained at least one nonevent at baseline, yet the FI remained unattainable because successive toggles moved the p-value away from the significance boundary rather than toward it, exhausting the arm before the threshold was crossed. Nonattainability is therefore not reducible to a zero-cell artifact: the majority of cases arise from the directional behavior of the Fisher exact p-value under the Walsh toggling path and would not be resolved by continuity corrections or exclusion of zero-cell tables.

Empirical dataset

In the empirical dataset, of the 143 total trials, 82 met the FI evaluation criterion for baseline significance with untied event counts. Of these, 80 had attainable FI, and two had unattainable FI, yielding an empirical nonattainability rate of 2.4% (2/82). The two unattainable cases were {910, 1262, 1028, 1646} (N = 4,846) and {1594, 638, 282, 58} (N = 2,572), confirming that nonattainability is not restricted to small tables.

Mechanisms of nonattainability

Across the full enumeration through N = 60, a total of 28,982 unattainable table instances were identified (cumulative across all evaluated sample sizes). Two distinct mechanisms accounted for all the cases.

In 12,278 instances (42%), the arm selected for FI toggling already contained zero nonevents at baseline. No allowable move existed, and the algorithm could not begin.

In 16,704 instances (58%), at least one legal toggle was available, but the permitted FI path moved Fisher's exact p-value farther from the nonsignificance boundary (0.05) rather than closer to it. The available toggle room was exhausted before the threshold was reached.

Both mechanisms reflect the same underlying constraint: the FI arm selection rule targets the arm with fewer absolute events, regardless of relative event rates or available toggle room.

Within the full enumerated range (N = 2-60), allocation imbalance showed a modest association with nonattainability (Spearman r = 0.324, 95% CI 0.321-0.327, p < 0.001), whereas total sample size showed only a negligible association (Spearman r = 0.015, 95% CI 0.011-0.019, p < 0.001).

## Discussion

The FI is not universally attainable across all valid clinical trial results. The mathematical proof establishes that nonattainability is possible; the enumeration shows that it is recurrent, and the empirical dataset confirms that it occurs in published clinical trials. Together, these three layers of evidence demonstrate that FI nonattainability is not a theoretical artifact but a structural property of the algorithm.

Although the existence of even a single valid table where FI is not attainable proves the theorem, the complete enumeration further strengthens the theorem by showing that nonattainability is not confined to a single unusual example. Unattainable cases first appear at N = 18 and recur at every larger sample size examined through N = 60. The mechanistic analysis adds a further dimension: in the majority of unattainable cases across the enumerated range, at least one legal toggle was available, but the permitted FI path moved Fisher's exact p-value farther from the nonsignificance boundary rather than closer to it. The arm-selection rule - targeting fewer absolute events - does not account for relative event rates or available toggle room.

The enumeration also showed that allocation imbalance, operationalized as the ratio of arm sizes (n1/n2), was modestly associated with nonattainability (Spearman r = 0.324), whereas total sample size was not (r = 0.015). This pattern is mechanistically consistent with the arm-selection rule: imbalance directly determines which arm is targeted and how much toggle room remains. The correlation was computed across the full enumerated range; within-N stratification was not performed, and defining a threshold of practical concern for the magnitude of imbalance requires data beyond this study.

The empirical results provide real-world confirmation. In this dataset, two of the 82 trials with significant baseline evaluations had unattainable FIs. That proportion should not be overinterpreted as a population prevalence estimate, as it is a convenience sample, but it confirms that nonattainability is not a purely theoretical concern.

A central implication follows. Calls for inclusion in routine statistical reporting rest on the premise that FI is computable for every statistically significant two-arm trial. That assumption is false. Valid baseline-significant trials exist for which the algorithm yields no finite result. The phrase “not attainable” is therefore not a marginal implementation detail; it reflects a genuine failure in the analysis of valid inputs to numeric outputs. The practical consequence is not simply that some trials obtain no FI value. The deeper problem is that exactly when the FI fails is not clearly defined. This means that any summary of FI across the literature is computed on an unknown subset of the evidence base, with trials excluded for poorly defined reasons unrelated to their raw outcomes.

This property is also distinct from standard statistical uncertainty. Fisher's exact test remains defined for these tables. The tables themselves are valid. Failure occurs because the FI algorithm imposes a constrained, discrete path through the table space and, for some inputs, that path either does not exist or does not reach the significance boundary, regardless of the number of steps taken.

The practical stakes of these findings are direct. Proposals for routine integration of FI into clinical trial reporting - including explicit recommendations that FI be included in guideline development - presuppose that every valid trial can receive a fragility value [11]. A mandatory reporting standard built on a metric that is not universally attainable would either silently exclude a subset of trials or require ad hoc handling of nonattainable cases with no standardized solution, introducing inconsistency into the very literature the standard is meant to organize [21].

Existing FI calculators handle nonattainability inconsistently. On the {3,0,4,11} counterexample, the Fragility Metrics Toolkit explicitly returns 'Not Attainable,' surfacing the failure mode for the user. ClinCalc returns a generic error message stating that an insignificant p-value could not be obtained during the calculation process, without naming the structural cause. This asymmetry compounds the silent-exclusion problem: investigators relying on tools that fail without explanation may attribute the result to a data error rather than to a structural property of the metric.

Two major cardiology trials, as well as an oncology trial, clearly illustrate this problem. FINEARTS-HF and FRESCO were identified post-hoc as illustrative large-trial examples; ISCHEMIA was one of the two unattainable cases in the prospective empirical dataset. In FINEARTS-HF, 910/2172 subjects in the low left ventricular ejection fraction (LVEF) group had adverse events, versus 1028/2674 in the 50-60% LVEF group (two-sided Fisher exact p = 0.016) [22]. This result yields an unattainable FI due to the structural algorithmic failure documented here. This major cardiology trial, because of an unattainable FI, would thus be excluded from meta-analyses that required inclusion of the FI. In the ISCHEMIA trial, comparing conservative treatment with open coronary artery bypass grafting, there were 1594/2232 subjects free from angina at 48 months in the conservative strategy compared with 282/340 in the bypass group (two-sided Fisher's exact p < 0.001). These results once again yield an unattainable FI [23]. A third concrete example is the FRESCO trial [24], where deaths occurred in 188/278 in the treatment arm, and 109/138 in the placebo arm (two-sided Fisher's exact p = 0.016). Again, the FI is unattainable in this study, thereby excluding it from meta-analyses and systematic reviews that examine statistical fragility. Calls for mandatory inclusion of a statistical fragility metric that silently excludes trials of this scale have major negative consequences. Readers will have no way of knowing which trials were omitted or whether their inclusion would have altered the evidence synthesis.

Active research into updated and alternative fragility metrics reflects recognition of the structural limitations of the original FI definition. The Lin and Chu R package, for example, allows event status modifications in both treatment arms rather than restricting toggling to the arm with fewer events, avoiding one source of nonattainability [15]. The global FI increases available toggle moves, permitting cell-to-cell reallocation in any direction across the entire table, thereby avoiding independent arm-level constraints altogether [25]. Whether these alternatives are themselves universally attainable is a separate question requiring formal analysis. The present findings establish only that the original FI is not. Whatever direction the field pursues, the core requirement is the same: a fragility metric used as a universal reporting standard must provide a value for every trial it evaluates. The present findings provide the formal basis for that transition.

Limitations

In this study, only 2 × 2 tables analyzed with a two-sided Fisher's exact test at alpha = 0.05 were examined. This scope is appropriate because the FI was originally defined under exactly these conditions, so the evidence of nonattainability applies specifically to the conditions under which the metric was designed. Tied-event tables were excluded because the FI definition does not provide a tie-breaking rule; this exclusion is a limitation of the metric itself rather than of the analysis. The empirical dataset was not a systematic review, and the 2.4% nonattainability rate should not be interpreted as a population prevalence estimate; however, the two empirical cases occurred in large trials (N = 4,846 and N = 2,572), confirming that nonattainability is not an artifact of small-sample table geometry. The enumeration extended through N = 60 only, but this is sufficient: the theorem is established by counterexample alone, and the enumeration serves to demonstrate recurrence rather than to determine a prevalence ceiling. As this is a single-author study, there was no independent dual-verification of the enumeration results; however, the complete enumeration code is archived at Zenodo (DOI: 10.5281/zenodo.18916363) and is openly available for independent replication.

## Conclusions

The FI is not universally attainable. Valid baseline-significant 2 × 2 tables exist for which no finite FI can be computed under the metric's own algorithmic rules. Complete enumeration shows that these cases recur across the valid table space, and empirical analysis confirms their occurrence in published clinical trials. This nonattainability is a formal property of the FI algorithm itself.
